# Multiplex Ligation-Dependent Probe Amplification for Simultaneous Identification of *Bungarus multicinctus* and Its Common Adulterants in a Single Assay

**DOI:** 10.3389/fphar.2020.00501

**Published:** 2020-04-21

**Authors:** Yuxin Zhou, Jing Nie, Shiqi Yu, Zhigang Hu, Bo Wang

**Affiliations:** ^1^College of Pharmacy, Hubei University of Chinese Medicine, Wuhan, China; ^2^Chinese Medicine Testing and Research Center, Hubei Institute for Drug Control, Wuhan, China

**Keywords:** *Bungarus multicinctus*, multiplex ligation-dependent probe amplification, adulterant identification, simultaneous identification method, traditional Chinese medicine

## Abstract

*Bungarus multicinctus*, an important traditional Chinese medicine, possesses remarkable medicinal activities, while lots of adulterants from other species were misused as *B. multicinctus* for its large demand and resource starvation. In order to accurately identify *B. multicinctus* and its common adulterants such as *Sinonatrix annularis*, *Xenochrophis flavipunctatus*, *Deinagkistrodon acutus*, and *Naja atra*, a simultaneous identification method was designed with multiplex ligation-dependent probe amplification (MLPA) analysis. Five species-specific MLPA probe-couples for *B. multicinctus* and its common adulterants were designed based on the universal primer amplified *COI* sequences, which can specifically detect the five species with no mutual interference, and sensitivity analysis showed as less as 5% *B. multicinctus* or 8.75% adulterants in the mixed samples can be identified in a MLPA assay, especially, the relative quantity of the adulterants can be also inferred based on the MLPA peak area values. Moreover, the results of the present study confirmed the effectiveness of this technique in terms of simultaneous identification of *B. multicinctus* and its common adulterants in an assay, which has great potential for ensuring the safety of this commercially valuable snake species.

## Introduction

According to the reptile database, there are 3,701 kinds of snake species in the world, 286 of which are distributed in China (Yanqing et al., 2019) and about the one tenth are frequently used for medicinal and commercial purposes, for example, snake venom and tissues are used to treat diseases, skins are used in the luxury leather goods industry (Singh et al., 2012; Murray-Dickson et al., 2017). In China, 17 snake species were recorded in the world-famous medical book Compendium of Materia Medica (Pipeng et al., 2013), and there are currently still five snake species included in the Chinese Pharmacopoeia (2015 Edition) as statutory provisions for traditional Chinese medicine. *B. multicinctus*, belonging to the Elapidae family, is a famous statutory medicinal snake species with effect of dispelling wind and removing obstruction in the meridians, and it was also used as a chief ingredient in many polyherbal formulations for the treatment of cancer (Wuguang et al., 2017; Linchun et al., 2018). However, due to the low reproductive capacity of *B. multicinctus* and the habitat destruction, the survival of this snake species had been threatening, which exerted a detrimental impact on ecosystems and biodiversity. Furthermore, based on its good effect in treating rheumatoid arthritis, epilepsy, urticaria, and cervical spondylosis (Jinting, 2008), this officinal snake is in huge demand, then, artificially propagated *B. multicinctus* and its counterfeits were gradually appeared in the medicine market (Tingping, 2001). For the purpose of protecting of species diversity and monitoring the safety of the medical market, it is necessary to develop methods for distinguishing this species.

For resource protection and drug safety, it is generally believed that the first step is to accurately identify species. The morphological characteristics of *B. multicinctus* and some other processed snakes (Chao et al., 2015), such as *D. acutus*, *N. naja* (Yiquan et al., 2000), are very similar. So, morphological examination can´t successfully be employed without visually recognizable features or has been otherwise altered in those species (Yanqing et al., 2019; Yi et al., 2017). In this regard, molecular identification techniques have been proven to be an effective tool for species identification. Although some interesting molecular biology techniques, such as sequence characterized amplified region (SCAR) (Yau et al., 2002) and polymerase chain reaction (PCR) (Jingxue et al., 2010) have been applied to snake identification, these methods can distinguish the authenticity of snake species but not perform simultaneous identification and relative quantitative analysis. In the world, one fifth of snakes are poisonous, most of them belong to Viperidae, Elapidae, Colubridae, and Atractaspididae families (Chi-Hsin et al., 2016). In clinical practice where the snakes are often swallowed by powder, misuse or mixed use of these poisonous snakes will bring hidden dangers to patients.

The multiplex ligation-dependent probe amplification (MLPA) (Schouten et al., 2002) as an emerging molecular diagnostic technique can detect the difference in nucleic acid sequences with a small amount of DNA and possess capability of detecting up to 50 genomic DNA sequence difference in one reaction relied on DNA denaturation, hybridization, ligation, and PCR reaction (Os and Schouten, 2011; Samelak-Czajka et al., 2017). Recently, the technology has been applied to the clinical diagnosis of diseases, such as Duchenne muscular dystrophy (Guertler et al., 2014), Gaucher disease (Schouten et al., 2002), Acute leukemia (Reyes-Núñez et al., 2017), and it also has been received wide attentions in the field of allergen detection (López-Calleja et al., 2016), genetically modified species identification (Holck et al., 2009), and herb identification (Bo et al., 2018).

In the present study, *B. multicinctus* and the four species usually acted as its adulterants were studied. Five species-specific MLPA probe-couples were designed based on the mitochondrial amplification sequences of the five snake species. The specificity of the probes was verified by hybridization of single probe or probemix to the DNA target, and the proportion of the adulterants in *B. multicinctus* was estimated by the MLPA peak areas obtained from the capillary electrophoresis analysis. The current study provided a method for simultaneous identification and relative quantification of the adulterants in this important valuable snake, and also provided a reference for adulterants identification in other medicinal species.

## Materials and Methods

### Sample Material and DNA Extracted

These samples, including *B. multicinctus*, *S. annularis*, *X. flavipunctatus*, *D. acutus*, and *N. atra* were collected Guangxi, Yunnan, Hubei, Hunan, Jiangxi, and Zhejiang Province, and identified by Dr. Bo Wang who works in the area of species authenticity and certification in Hubei Institute for Drug Control. The voucher specimens were deposited at the Chinese medicine specimen museum of Hubei Institute for Drug Control and Institute of Chinese Materia Medica China Academy of Chinese Medical Sciences, the sample numbers in two institutions were: HBYJ2016101- HBYJ2016125, and 1040412001-1040412025 respectively. The species identification was confirmed using the COI universal barcode sequence. The total DNA of these samples were extracted using animal tissue genomic DNA kit (Zoman Biotechnology Co., Ltd., Beijing, China) as described by the manufacturer’s instruction. The concentration of DNAs was estimated using ND-2000 spectrometer (Nanodrop Technologies, Wilmington, DE, USA). DNAs were stored at −20°C for further analysis. It is worth mentioning that the collection of all samples studied in this experiment was in compliance with relevant legal provisions, and we required only a small tissue sample, which does not affect snake resources. The protocol was approved by Institutional Animal Care and Use Committee affiliated with the Hubei Provincial Academy of Preventive Medicine & Hubei Provincial Center for Disease Control and Prevention.

### Hybrid Sequence Screening and Phylogenetic Reconstruction

A pair of universal primers (Forward primer: 5′-TATTCTCAACTAACCACAA AGA-3′; Reverse primer: 5′-ACTTCTGGTTGACCAAAGAATCA-3′) was screened to amplify the DNA fragments from the five species. PCR amplification was performed in a total volume of 25 µl containing 21 µl of PCR Mix (TsingKe, Beijing, China), 1 µl of forward primer (10 µM, Sangon), 1 µl of reverse primer (10 µM, Sangon), 2 µl of total DNA. Thermal cycling was performed in a Bio-Rad thermocycler (Bio-Rad, California, CA, USA) using a PCR process: 94°C for 3 *min*; 40 cycles of 94°C for 40 s, 48.5°C for 30 s, and 72°C for 60 s; 72°C for 7 *min*, and subsequent storage at 4°C. The amplified fragments were sequenced by the ABI3730XL sequencer (Applied Biosystems Co., Shanghai, China). Sequencing data were processed by the CodonCode Aligner V3.7.1 (CodonCode Co., Centerville, MA, USA). Phylogenetic relationship was analyzed by the neighbor-joining (NJ) method in the MEGA7 program (National Institutes of Health, Bethesda, MD, USA).

### MLPA Probe Design

The species-specific MLPA probes for B. multicinctus, S. annularis, X. flavipunctatus, D. acutus, and N. atra were designed base on the COI sequences amplified by the universal primers, and were referenced to the suggestions of MRC-Holland (Amsterdam, Netherlands; http://www.mrc-holland.com). In order to find the suitable regions for designing the species- specific probes, the sequences from the five species were aligned and analyzed in the NCBI database (http://www.ncbi.nlm.nih.gov). The LPO and RPO folding and ΔG were tested by mfold Web Server (http://unafold.rna.albany.edu/?q=mfold) to choose the optimal probes. In order to avoid interfering cross-reactivities, Blast (https://blast.ncbi.nlm.nih.gov/Blast.cgi) was used to evaluate the specificity of five species-specific MLPA probe-couples.

### MLPA Assay

All steps of the MLPA reactions were performed in thermocycler (Bio-Rad, California, CA, USA) using the SALSA MLPA EK1 reagent kit (MRC-Holland, Amsterdam, Netherlands) according to manufacturer’s instruction. All components were included in the SALSA MLPA EK1 reagent kit, except DNA samples, ddH_2_O, probes, and TE buffer. Routinely, 5 µl of DNA sample (50–100 ng) was denatured at 98*°*C for 5 *min*. A non-template control with 5 µl of TE buffer was included to reveal possible reagent contaminations. After all samples were cooled to 25*°*C, removed from the thermocycler to add 3 µl hybridization mix (1.5 µl SALSA MLPA buffer and 1.5 µl probes) and heated at 95*°*C for 1 *min*, 60*°*C for 16 *h*. For the ligation reaction, 32 µl ligase mix (25 µl ddH_2_O, 3 µl ligase buffer A, 3 µl ligase buffer B, and 1 µl Ligase-65 enzyme) were added and incubated for 15 *min* at 54*°*C. Subsequently, the enzyme was inactivated by heated at 98*°*C for 5 *min*. Amplification of the ligation products was carried out with the universal SALSA PCR primers by adding 10 µl polymerase mix (7.5 µl ddH_2_O, 2 µl SALSA PCR primer mix, and 0.5 µl SALSA polymerase) and implemented the PCR reaction at 35 cycles of 95*°*C 30 s, 60*°*C 30 s, 72*°*C 1 *min*; 72*°*C 20 *min*. PCR products were stored in the dark at 4*°*C until further analysis. To assess MLPA reproducibility, three replicates of each DNA extract were analyzed in each experiment.

### Capillary Electrophoresis

For each reaction, 1 µl PCR product, 9 µl HiDi formamide, and 0.5 µl Genescan 500 LIZ size standard were mixed. The samples were denatured at 95°C for 5 *min* and then cooled on ice. Collected fragment data were performed 3130xl Genetic analyser (Applied Biosystems) using the following settings: run temperature: 60 °C; injection voltage: 1.2 kV; injection time: 16 s; run voltage: 15 kV, run current: 5 µA, and run time: 1800 s. The size, peak height, and peak area of fluorescent PCR fragments were extracted by GeneMaeker (SoftGenetics, Pennsylvania, PA, USA) and used for quantitative analysis.

### Specificity and Sensitivity of MLPA

The sample of *B. multicinctus* and its common adulterants were used to assess the specificity of the MLPA test, which we tested by DNA hybridized with single probe and probemix, respectively. The sensitivity of MLPA was assessed by change the amount of DNA from 200ng to 2.5ng.

### Detection of Mixed Samples

To detect *B. multicinctus* and its common adulterants in a mixed sample, we mixed *B. multicinctus* and the four common adulterants into different proportions as composite samples. The ratio of *B. multicinctus* was 65%, 50%, 30% and 5%, respectively. After capillary electrophoresis analysis, we can visually observe the actual proportion of the five species in the composite sample.

## Results

### Identification Sequence Analysis

**Figure 1:**
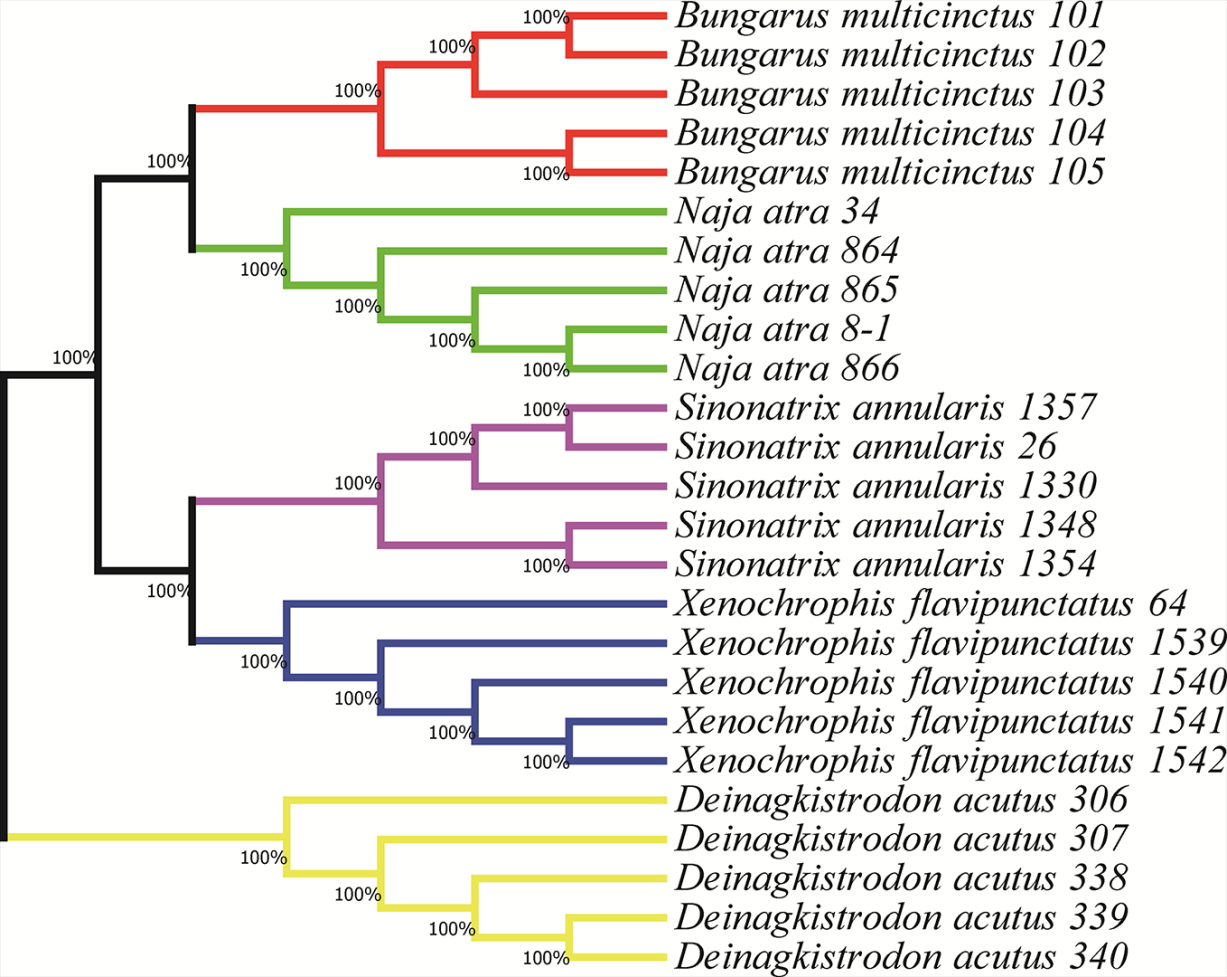
Phylogenetic tree constructed using neighbor joining (NJ), based on the sequences amplified by universal primers from five species.

In order to ensure the accuracy and reproducibility of the assay, a NJ tree of the five species was constructed using the snake sequences amplified by the universal primers (Limpiti et al., 2014) (**Figure 1**). As can be seen from the NJ tree, each species was clustered into one branch, and the support rate was 100%. It was strongly supported that the position of *S. annularis* and *X. flavipunctatus* as a sister of the close related species in the Colubridae. For the same reason, both *B. multicinctus* and *N. atra* belonging to Elapidae were clustered together. This result indicated that the sequences amplified by the universal primers can be used to distinguish five species and design the species-specific probes for next MLPA analysis. Based on this, the species-specific MLPA probes toward the five snake species were designed, and were shown in **Table 1**.

**Table 1:** The information of probes in this MLPA assay.

Species	Probe name	Sequence(5*′*→3*′*)	Fragment size	Calculated size
*B. multicinctus*	LPO	**GGGTTCCCTAAGGGTTGGA**TACCAATCATAATCGGAGGGTTTGGCAACT	102	101
	RPO	GACTTATCCCTTTAATAATCGGGGCCCCTG**TCTAGATTGGATCTTGCTGGCAC**		
*D. acutus*	LPO	**GGGTTCCCTAAGGGTTGGA *T*** ATCTTTTCTCTCCACTTAGCCGGGGCATCCTCTA	112	110
	RPO	TCCTAGGGGCAATTAACTTCATCACTACGTGCATC**TCTAGATTGGATCTTGCTGGCAC**		
*N. atra*	LPO	**GGGTTCCCTAAGGGTTGGA *G*** TGCCTAAGCATACTAATACGCATAGAACTGACCCAGCCCGGATCACTA	139	137
	RPO	TTCGGCAGTGACCAGATCTTCAACGTACTTGTAACTGCCCACGCATTT**TCTAGATTGGATCTTGCTGGCAC**		
*S. annularis*	LPO	**GGGTTCCCTAAGGGTTGGA *T*** ACAGTGTACCCGCCATTGTCTGGAAAC	97	95
	RPO	CTAGTACATTCAGGCCCATCGGTGGAC**TCTAGATTGGATCTTGCTGGCAC**		
*X. flavipunctatus*	LPO	**GGGTTCCCTAAGGGTTGGA *T*** TACCCATCATGATCGGTGGTTTCGGGAACTGA	107	105
	RPO	CTAATCCCCCTCATACTAGGAGCCCCCGACAT**TCTAGATTGGATCTTGCTGGCAC**		

Uppercase bold: Primer binding sequence; Uppercase: Hybridizing sequence; Uppercase italics: Stuffer base. All RPOs are 5′-phosphorylated.

### Evaluation of MLPA Specificity

**Figure 2:**
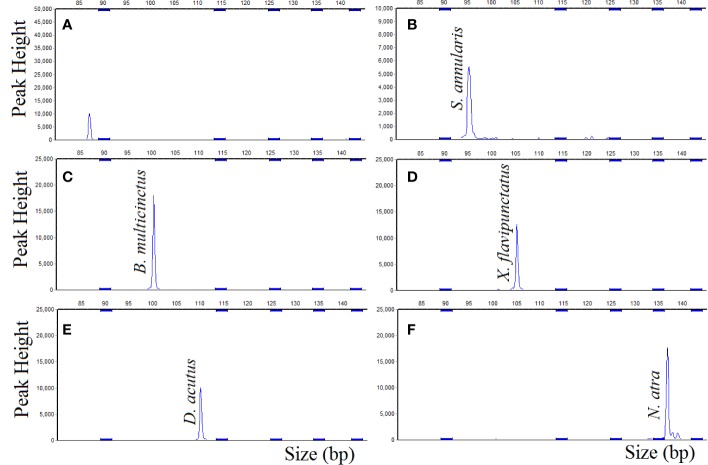
MLPA single probe specificity, DNA target hybridized with single probe. **(A)** negative control; **(B)** *S. annularis*; **(C)** *B. multicinctus*; **(D)** *X. flavipunctatus*; **(E)** *D. acutus*; **(F)** *N. atra*.

The complete hybridization sequence (left hybridizing sequence and right hybridizing sequence) for each probe was aligned with the Blast (https://blast.ncbi.nlm.nih.gov/Blast.cgi), and each probe was only matched to a unique species, which theoretically excluding cross-contamination. To assess the specificity of the probes, the single probe and probemix were hybridized to the DNA samples for 16 h, respectively. The size of amplification fragments which hybridization of the DNA target and the single probe were in line with the expected size, *S. annularis* 95 nt, *B. multicinctus* 101 nt, *X. flavipunctatus* 105 nt, *D. acutus* 110 nt, and *N. atra* 137 nt (**Figure 2**). The DNA target of the five species were hybridized with the single probe, the actual length of amplicons were differed from the designed length by 1 to 2 nt, which was due to the different mobility of DNA and/or the distinct labeling of the standard and the amplified fragments (García-García et al., 2017).

**Figure 3:**
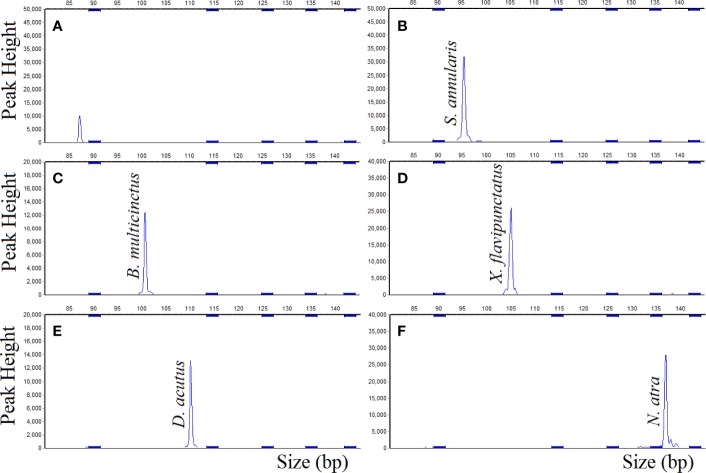
MLPA probemix specificity, DNA sample hybridized with probemix. **(A)** negative control; **(B)** *S. annularis*; **(C)** *B. multicinctus*; **(D)** *X. flavipunctatus*; **(E)** *D. acutus*; **(F)** *N. atra*.

The results of DNA target hybridized with the probemix (**Figure 3**) were similar to the results that DNA target hybridized with the single probe. The corresponding probes gave positive signal for *S. annularis, B. multicinctus, X. flavipunctatus, D. acutus*, and *N. atra*, respectively, as expected, which was also stable in gradually increased hybridization temperature from 60°C to 68°C with a gradient of 1°C. The results showed that the MLPA probes exhibit strong specificity and reproducibility toward in five species.

### Evaluation of MLPA Sensitivity

To verify the ability of MLPA method, five species of snakes were detected in order to promote the application of this technology in traditional Chinese medicine. The DNA concentration of the *B. multicinctus* and its common adulterants was adjusted to 20 ng/µl before evaluating the sensitivity. Five targets were hybridized with probemix, changing the amount of total DNA samples from 200 ng to 50 ng, all signal peaks were with the expected size, which indicated that the five species could be detected simultaneously even if change the amount of DNA samples. The amount of *B. multicinctus* DNA sample (100 ng, 80 ng, 60 ng, 32.5 ng, 25 ng, 15 ng, 10 ng, 5 ng, 2.5 ng) and the amount of adulterant DNA sample (80 ng, 47.5 ng, 35 ng, 25 ng, 23.75 ng, 17.5 ng, 11.785 ng, 8.75 ng, 6.25 ng, 4.375 ng) were changed until the amount of *B. multicinctus* DNA and adulterant DNA were reduced to 2.5 ng and 4.375 ng, respectively, the signal peak of the sample could be detected. The DNA extraction method in this experiment can obtain 10 to 30 **µ**g total DNA from 30 mg sample tissue. In order to obtain 2.5 ng total DNA, only 0.0025 ~0.0075 mg samples were required, indicating that the method requires very little sample. Thus the MLPA method can be used to identify *B. multicinctus* and its common adulterants in the traditional Chinese medicine market.

### Quantitative Adulterations of *B. multicinctus* Using MLPA Analysis

**Figure 4:**
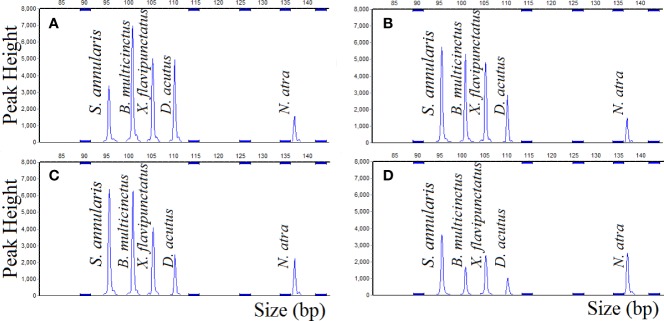
MLPA method simultaneously identifies, differentiates, and relatively quantifies five species **(A)** *B. multicinctus* accounts for 65%; **(B)** *B. multicinctus* accounts for 50%; **(C)** *B. multicinctus* DNA sample accounts for 30%; **(D)** *B. multicinctus* DNA sample accounts for 5%.

After MLPA detected the composite samples, the sizes and peak areas of the amplified fragments from the five species were directly obtained from capillary electrophoresis, indicating that five species could be simultaneously identified, differentiated, and relatively quantified by MLPA. The ratio of *B. multicinctus* in the composite sample was 65%, 50%, 30%, 5%, and the peak area ratio of *B. multicinctus* was 31.8%, 25.4%, 14.8% and 14.6%, indicated that with the decrease of *B. multicinctus* DNA target, the peak area of *B. multicinctus* was decrement. Comparing the ratio of *B. multicinctus* in the composite samples (theoretical ratio) with the peak area ratio of *B. multicinctus* (actual ratio), it could be found that when the theoretical ratio of *B. multicinctus* was more than 30%, the theoretical ratio was nearly twice the actual ratio (**Figure 4**). The concentration of the DNA samples has been adjusted before the experiment, however, the actual ratio was different from the theoretical ratio possibly due to the different copy number of the five species. When *B. multicinctus* accounted for 65% of the total DNAs and the four adulterants for 8.75%, five species could be detected simultaneously by MLPA method (**Figure 4A**). After changing the amount of *B. multicinctus* to 5%, signal peak of *B. multicinctus* was still detected (**Figure 4D**). This means that when *B. multicinctus* accounted for more than 30%, the proportion of the adulterants can be inferred and when the ratio of *B. multicinctus* was less than 30%, it can be determined whether the adulterants exist. As a result, the developed MLPA method for *B. multicinctus* and its common adulterants detection is prompt and reliable for situations where *B. multicinctus* may be contaminated by its common adulterants.

## Discussion

In this study, we investigated the usefulness of the MLPA method as a prompt and effective way to detect *S. annularis, X. flavipunctatus, D. acutus*, and *N. atra*, as common adulterants of *B. multicinctus*. Previous studies have shown that when morphological identification and PCR-based assay are used to authenticate *B. multicinctus* and its common adulterants (Chengyong, 2002), the results either subjective, inconspicuous, relies on experience, or expensive, time and resource consuming. MLPA, combine the characteristics of DNA probe hybridization and PCR technology, is an easy-to-learn, fast, high throughput, reliable, and low-cost technology. MLPA assay can be performed with thermocycler and capillary electrophoresis system. The MLPA probes are very simple, the base at the left probe 3*′*-terminal base and ligase-65 enzyme ensure the specificity of the ligation reaction, the right probe 5*′*-terminal base phosphorylation promote the ligation reaction (Cornelia and Suvi, 2012). Both left probe and right probe can be quickly obtained by synthesis, greatly reducing the test period (Stern et al., 2004). Probe hybridization sequences are short (50~70 nt) (Schouten et al., 2002). It is possible that this method can be used to detect highly fragmented DNA sample. With this advantage, MLPA can be used to detect samples that have been stored for many years, processed and DNA-degraded. The amount of DNA is small, such as research congenital adrenal hyperplasia (CAH) by MLPA, DNA concentration down to 0.3 ng/µl (1.6 ng total) (Karina Meden et al., 2008). In MLPA, a pair of primers amplified all species sequences, and therefore, the amplification efficiency of the sequences was same or similar. Despite the many advantages of the MLPA reaction, this method does have several non-fatal drawbacks. First, we recognized that the MLPA method avoids complicated steps, such as cell culture in medical diagnosis and significantly shortens the diagnosis period, but the long hybridization time is a disadvantage in identification of traditional Chinese medicine. In this work, we referred previous study to shorten the hybridization time to 3 h when evaluating the specificity of MLPA probes (Guertler et al., 2014), and each assay was repeated three times with expected and stable results. When analyzing the relative quantification of the five species in the composite samples, the hybridization time was extended to 16 hours in order to ensure sufficient hybridization of the DNA target and the probemix. However, it has been verified that shortening hybridization time to 30 min can still ensure the stability of the experiment, and the results can be obtained within 6 hours (Saxena et al., 2015). Second, it is important to select proper barcode marker and design the probe. Proper barcode marker should have the feature that effectively distinguish the species involved, and are based on common barcode markers like *ITS2*, *COI*, etc.

There are various forms of counterfeit in traditional Chinese medicine. Any medicine that is in large demand and high price has the possibility of adulteration. Common adulterations species were similar in genetic relationships or morphological characteristics. Some adulterants were mixed into traditional Chinese medicine in the form of power. How to accurately identify and quantify adulteration products has become a major problem. DNA-based identification technology was more stable than other material-based identification technologies for its higher stability, cell uniformity, and polymorphism (Sultana et al., 2018). Mitochondria are autonomous organelles in eukaryotes, mtDNA has high rate of evolution, stable amplification, and repeatability. It is rich in genetic differences among different species and is suitable for species identification among order, family, genus, and species (Meng et al., 2018). In the present study, the *COI* sequence, a gene in mtDNA, was selected to differentiate the five species. After we obtained two sets of species-specific probe from five species, we tested the specificity and determined that only one set could be used for this study.

Compared with identification methods in the previous studies, the highlights of MLPA are the advantages in both simultaneous identification of *B. multicinctus* and its common adulterants that are *S. annularis*, *X. flavipunctatus*, *D. acutus*, and *N. atra*, and relative quantitative analysis of the sample. Comparing the method used to identify *B. multicinctus* indicated that MLPA is a superior process because of easy-to-learn, fast, high throughput, reliable, and low-cost. With the improvement of material living standards, people´s attention on the safety of traditional Chinese medicine is increasing, and the governmental agencies must take effective measures to regulate the safety of traditional Chinese medicine. Our study results indicate that the MLPA method can accurately identify the authenticity of traditional Chinese medicine and ensure the safety of consumers.

## Conclusions

Our findings showed that the species-specific MLPA probes can distinguish the snakes including *B. multicinctus* and its common adulterants that is *S. annularis*, *X. flavipunctatus*, *D. acutus*, and *N. atra*. A MLPA analysis method was proposed for the identification, differentiation, and relative quantification of these snake species, which proved to be a very sensitive tool for the simultaneous identification of snake genotypes. This method was sensitive enough to detect as less as 5% *B. multicinctus* or 8.75% adulterant in mixed samples, revealing its adequacy as a simple and fast approach for species simultaneous identification. Thus, this technology has potential for market detection and relative quantification of the adulterants in this important valuable snake, and also provided a reference for adulterants identification in other medicinal species.

## Data Availability Statement

All datasets generated for this study are included in the article/supplementary material.

## Ethics Statement

The animal study was reviewed and approved by the Hubei Provincial Academy of Preventive Medicine & Hubei Provincial Center for Disease Control and Prevention.

## Author Contributions

Formal analysis: YZ, SY. Methodology: ZH, BW. Project administration: JN, BW. Writing – original draft: YZ. Writing – review and editing: BW. All authors read and approved the submitted version.

## Funding

This research was funded by the National Natural Science Foundation of China (81603246), and the Foundation of Hubei Food and Drug Administration (201801001).

## Conflict of Interest

The authors declare that the research was conducted in the absence of any commercial or financial relationships that could be construed as a potential conflict of interest.
